# The Association of Prenatal Vitamin D Status With Pregnancy and Neonatal Outcomes

**DOI:** 10.1210/jendso/bvad142

**Published:** 2023-11-20

**Authors:** Di Mao, Lai-Yuk Yuen, Chung-Shun Ho, Chi-Chiu Wang, Claudia Ha-Ting Tam, Michael Ho-Ming Chan, William L Lowe, Ronald Ching-Wan Ma, Wing-Hung Tam

**Affiliations:** Department of Obstetrics and Gynecology, The Chinese University of Hong Kong, Hong Kong SAR, China; Department of Obstetrics and Gynecology, The Chinese University of Hong Kong, Hong Kong SAR, China; Department of Chemical Pathology, The Chinese University of Hong Kong, Hong Kong SAR, China; Department of Obstetrics and Gynecology, The Chinese University of Hong Kong, Hong Kong SAR, China; Li Ka Shing Institute of Health Sciences, The Chinese University of Hong Kong, Hong Kong SAR, China; School of Biomedical Sciences, The Chinese University of Hong Kong, Hong Kong SAR, China; Department of Medicine and Therapeutics, The Chinese University of Hong Kong, Hong Kong SAR, China; Department of Chemical Pathology, The Chinese University of Hong Kong, Hong Kong SAR, China; Department of Medicine, Northwestern University Feinberg School of Medicine, Chicago, IL 60611, USA; Li Ka Shing Institute of Health Sciences, The Chinese University of Hong Kong, Hong Kong SAR, China; Department of Medicine and Therapeutics, The Chinese University of Hong Kong, Hong Kong SAR, China; Hong Kong Institute of Diabetes and Obesity, The Chinese University of Hong Kong, Hong Kong SAR, China; Department of Obstetrics and Gynecology, The Chinese University of Hong Kong, Hong Kong SAR, China

**Keywords:** vitamin D, maternal, umbilical cord, placental transfer, pregnancy outcomes, neonatal outcomes

## Abstract

**Context:**

Vitamin D inadequacy is globally prevalent among pregnant women; however, its impact on pregnancy remains inconclusive.

**Objective:**

This study aims to explore the associations of maternal and umbilical cord serum 25-hydroxyvitamin D (25(OH)D) levels with pregnancy and neonatal outcomes.

**Method:**

We used archived serum samples from the Hyperglycemia and Adverse Pregnancy Outcome (HAPO) Study participants in the Hong Kong center and assayed maternal 25(OH)D levels at midgestation and umbilical cord 25(OH)D at birth using liquid chromatography–tandem mass spectroscopy. Data regarding pregnancy and perinatal outcomes were extracted from the HAPO study dataset and the hospital computerized medical system.

**Results:**

Only 247 (16.4%) mothers and 66 (5.0%) neonates met the criteria for vitamin D sufficiency (ie, 25(OH)D ≥ 75 nmol/L). The ratio of umbilical cord to maternal vitamin D levels was positively associated with maternal age and ambient solar radiation at the month of delivery, while negatively associated with maternal serum total 25(OH)D at midgestation (all *P* < .001). Umbilical cord serum 25(OH)D was independently associated with a lower primary cesarean section rate (OR 0.990, 95% CI 0.982-0.999; *P* = .032). There were no associations of maternal and umbilical cord 25(OH)D levels with other adverse pregnancy and neonatal outcomes.

**Conclusion:**

Placental vitamin D transfer was found to be higher with a lower maternal vitamin D level, older maternal age, and higher ambient solar radiation at the time of the delivery. The protective effect of sufficient vitamin D in a cesarean section will require further studies.

Vitamin D is not only recognized for its role in the skeletal system, but also for its ability to regulate gene expression and its involvement in a wide range of chronic diseases [1]. Circulating 25-hydroxyvitamin D (25(OH)D) consists mainly of 2 forms, vitamin D3 (cholecalciferol), which is produced in the skin after sun exposure, and vitamin D2 (ergocalciferol), which is absorbed from food intake. Vitamin D insufficiency and deficiency have become a global health concern, especially among pregnant women who have greater requirements for calcium and vitamin D. Globally, vitamin D deficiency occurs in over 50% of pregnant women and infants at birth [2]. Several observational studies suggested an association of vitamin D deficiency with preeclampsia [3, 4], gestational diabetes mellitus (GDM) [5], preterm delivery [6], cesarean section, low birth weight, and small for gestational age (SGA) [6], but these findings remain inconclusive [7]. Despite vitamin D supplementation during pregnancy showing a convincing effect on reducing the risk of wheezing in offspring [8, 9], there is still a paucity of evidence to support a robust effect of vitamin D on preventing adverse pregnancy and neonatal outcomes. Heterogeneity among previous studies, including ethnicity, the definition of vitamin D deficiency, the extent of confounder adjustment, and the methodology for measuring 25(OH)D levels, may account for their divergent results [10]. Indeed, we previously reported that about 4% of research participants would be wrongly classified as having “optimal” vitamin D status if the assay method was unable to separate the inactive form of 25(OH)D [11].

This study aimed to assess the vitamin D status among pregnant women and neonates, and the factors influencing their vitamin D concentration. More importantly, we further examine whether prenatal 25(OH)D status is associated with adverse pregnancy and birth outcomes.

## Materials and Methods

### Study Cohort and Setting

The participants were ethnic Chinese mothers who participated in the Hyperglycemia and Adverse Pregnancy Outcome (HAPO) study between 2000 and 2006 at the Hong Kong study center. Details of the HAPO Study were described previously [11, 12]. All pregnant women booked for antenatal care in the study center were eligible to participate unless they met an exclusion criteria, namely, teenage pregnancy younger than 18 years of age, plan to undergo delivery at another hospital, an uncertain date of last menstrual period and no ultrasonographic estimate of gestational age between 6 and 24 weeks, inability to complete the oral glucose tolerance test (OGTT) within 32 weeks of gestation, multiple pregnancy, conception by means of assisted reproduction, glucose testing before recruitment, diagnosis of diabetes during the current pregnancy, or diagnosis of diabetes before the current pregnancy and requiring treatment with medication. All participants underwent a standard 75-g OGTT between 24 and 32 weeks of gestation. Demographic and clinical data were collected from dual data sources from standardized research questionnaires of the HAPO study and the electronic clinical medical system of the Hospital Authority. All participants provided written consent form to participate in the HAPO study and its ancillary studies. And the current study was approved by the Joint Chinese University of Hong Kong-New Territories East Cluster Clinical Research Ethics Committee.

### Blood Sample Collection

Maternal blood specimen were retrieved at the time of OGTT, and the umbilical cord blood samples were collected at delivery. Both maternal and umbilical cord serum specimen were stored at −80 °C at the Northwestern University Feinberg School of Medicine, Chicago, until they were shipped back on dry ice in a well-shielded container to Hong Kong for the 25(OH)D assays. Serum 25(OH)D is stable even up to 24 years when the samples are stored below −20 °C [13].

### Laboratory Assay

Serum 25(OH)D2 and 25(OH)D3 levels were measured using archived sera and a liquid chromatography–tandem mass spectrometry method that separated 3-epi-25(OH)D3, the biologically inactive form, from the vitamin D metabolites at the Biomedical Mass Spectrometry Unit, Department of Chemical Pathology, the Chinese University of Hong Kong. The details of the assay were previously described [11].

The lower limit of detection and limit of quantitation were 0.4 and 1.0 nmol/L, respectively. The analytical measurement ranges were 1 to 750 nmol/L for serum 25(OH)D2 and 25(OH)D3. Between-batch coefficients of variation of 25(OH)D2 and 25(OH)D3 were <3.8%. Accuracy performance was monitored regularly by measuring proficiency testing samples from the United Kingdom Vitamin D External Quality Assessment Scheme.

### Outcome Measures

The pregnancy outcomes included the rate of preeclampsia, preterm delivery, and primary cesarean section. The diagnosis of preeclampsia was made when systolic blood pressure was ≥140 mmHg and/or diastolic blood pressure ≥90 mmHg on 2 or more occasions a minimum of 6 hours apart and proteinuria of 1+ or more on a dipstick test or a urine protein level of 300 mg or more for a 24-hour period after 20 weeks of gestation was present [14]. Preterm birth was defined as delivery before 37 weeks of gestation.

The birth outcomes included SGA, large for gestational age (LGA), adiposity, Apgar score <7 at 5 minutes after birth, umbilical cord arterial pH <7.0, and admission to the neonatal intensive care unit. Neonatal measurements, including weight, length, and skinfold thickness at 3 sites (flank, subscapular, and triceps), were obtained in a standardized method by trained research staff within 72 hours of delivery as previously described in the original HAPO study [12]. SGA and LGA were defined with reference to local data on gestational age–specific birth weight percentiles obtained for boys and girls from a territory-wide study in Hong Kong [15]. Birth weight for gestational age z score for each neonate was calculated as (actual birth weight – the expected sex- and gestational age–specific birth weight)/the SD of expected birth weight. SGA was defined as the birth weight for gestational age z score below −1.28, while LGA as z score above 1.28. Adiposity referred to neonatal body fat mass ≥90th percentile. The infant’s body fat mass was estimated with the equation: 0.39055 (birth weight) + 0.0453 (flank skinfold) – 0.03237 (length) + 0.54657 as previously used in the HAPO study [16, 17]. Fat-free mass was calculated by subtracting fat mass from birth weight. Body fat mass was adjusted for gestational age and gender using linear regression prior to analyses. The percentage of body fat (ie, fat mass divided by birth weight) was used as a surrogate marker for body fat composition to quantify neonatal adiposity.

### Statistical Analysis

Maternal vitamin D status was categorized into severe deficient, deficient, insufficient, and sufficient, based on maternal total serum 25(OH)D (ie, sum of maternal serum 25(OH)D2 and 25(OH)D3 <25, 25-49.9, 50-74.9, and ≥75 nmol/L, respectively) according to the Endocrine Society definition [18]. The definition of neonatal vitamin D and severe vitamin D deficiency remains controversial; neonatal vitamin D status was categorized similarly to maternal vitamin D status for comparison of pregnancy and neonatal outcomes in this paper. The monthly global solar radiation recorded at the Hong Kong Observatory during the study period was obtained from the public domain at the website http://www.hko.gov.hk/en/index.html.

Data distribution was examined by histogram and data of skew distribution was log-transformed for normal distribution prior to analyses if appropriate. Continuous data were expressed as mean ± SD while categorical data were expressed as counts (percentage). Between group differences were compared by analysis of variance (ANOVA) for continuous variables and chi-square tests for categorical variables. Bonferroni correction was used for the post hoc analysis to investigate which pairs of groups had significantly different means after ANOVA results in a significant F-statistic. An adjusted *P* < .05 after Bonferroni correction indicated statistical significance for the mean difference. Linear regression was used to explore the association between maternal and neonatal 25(OH)D levels. Multivariate logistic regression analyses were used to show the associations of maternal and neonatal 25(OH)D with the pregnancy and birth outcomes. *P* < .05 was considered statistically significant. Statistical analyses were performed using IBM SPSS 26.0 software (SPSS Inc., Chicago, IL, USA).

## Results

### Maternal Characteristics, and Pregnancy and Neonatal Outcomes

A total of 1502 mothers and 1321 infants had serum total 25(OH)D levels available for analysis. Table 1 shows the maternal characteristics, pregnancy and neonatal outcomes with maternal serum vitamin D status; 247 (16.4%) were vitamin D sufficient. There were trends for increasing maternal age, parity, maternal 1-hour and 2-hour glucose levels and GDM rate and for decreasing maternal weight and primary cesarean section rate with higher maternal serum total 25(OH)D levels, but the rates of primary cesarean section due to common intrapartum indications like failure to progress and fetal distress were not significantly different from the vitamin D levels. Maternal serum total 25(OH)D level increased with the ambient solar radiation at the time of blood draw (*P* < .001). Umbilical cord serum total 25(OH)D level at birth also increased with maternal serum total 25(OH)D level (*P* < .001). However, the umbilical cord serum to maternal serum 25(OH)D ratio showed a decreasing trend with maternal serum levels (*P* < .001).

**Table 1.** Comparisons of maternal characteristics, pregnancy and neonatal outcomes by maternal serum total 25(OH)D level

	Maternal serum total 25(OH)D level (nmol/L)				*P*
	<25 (n = 32)	25-49.9 (n = 580)	50-74.9 (n = 643)	≥75 (n = 247)	
**Maternal characteristics at recruitment**					
Maternal age (years)	28.5 ± 4.0	30.0 ± 5.1	31.3 ± 4.6	31.7 ± 4.6	<.001
Parity					
0	25 (78.1)	393 (67.8)	389 (60.5)	126 (51.0)	<.001
1	7 (21.9)	153 (26.4)	206 (32.0)	102 (41.3)	
≥2	0	34 (5.9)	48 (7.5)	19 (7.7)	
Prepregnant weight (kg)	52.1 ± 8.3	52.0 ± 8.2	52.0 ± 7.3	50.9 ± 7.2	.21
Prepregnancy BMI (kg/m^2^)	20.5 ± 3.4	20.7 ± 3.0	20.7 ± 2.7	20.5 ± 2.7	.74
Smoker, n (%)	0	21 (3.6)	11 (1.7)	2 (0.8)	.03
**Maternal characteristics at OGTT**					
Gestational age at blood draw (week)	27.6 ± 1.4	27.6 ± 1.3	27.7 ± 1.2	28.0 ± 1.4	<.001
Maternal weight (kg)	61.7 ± 8.4	61.9 ± 8.9	61.2 ± 7.9	60.0 ± 7.5	.03
Maternal BMI (kg/m^2^)	24.3 ± 3.7	24.6 ± 3.1	24.4 ± 2.8	24.1 ± 2.7	.30
Fasting plasma glucose level (mmol/L)	4.32 ± 0.30	4.36 ± 0.31	4.37 ± 0.34	4.35 ± 0.36	.70
1-hour plasma glucose level (mmol/L)	6.93 ± 1.42	7.47 ± 1.60	7.69 ± 1.71	7.85 ± 1.71	.001
2-hour plasma glucose level (mmol/L)	6.05 ± 1.07	6.38 ± 1.18	6.61 ± 1.35	6.68 ± 1.34	<.001
AUC_glu_	727 ± 101	770 ± 125	790 ± 137	802 ± 136	<.001
HbA1c (%)	4.96 ± 0.16	4.92 ± 0.33	4.92 ± 0.37	4.94 ± 0.39	.80
GDM	1 (3.1)	61 (10.5)	99 (15.4)	38 (15.4)	.02
**Pregnancy outcomes**					
Gestational age at delivery (week)	39.2 ± 1.9	39.4 ± 1.5	39.3 ± 1.5	39.2 ± 1.4	.30
Preterm delivery <37 weeks	2(6.3)	26(4.5)	36(5.6)	13(5.3)	.83
Preeclampsia	0	11 (1.9)	19 (3.0)	3 (1.2)	.29
**Primary cesarean section**	9 (28.1)	138 (23.8)	114 (17.7)	43 (17.4)	.02
Indication of primary cesarean section					
Failure to progress	0 (0)	23 (4.0)	23 (3.6)	9 (3.6)	.10
Fetal distress	4 (12.5)	36 (6.2)	28 (4.4)	9 (3.6)	
Other causes	5 (15.6)	79 (13.6)	63 (9.8)	25 (10.1)	
**Neonatal outcomes**					
Baby's gender (male)	15(46.9)	290(50.0)	336(52.3)	131(53.0)	.76
Birth weight (g)	3082 ± 438	3184 ± 422	3149 ± 439	3174 ± 430	.34
Fat free mass (g)	2869 ± 239	2879 ± 279	2858 ± 289	2871 ± 287	.66
Fat mass (g)	313 ± 138	322 ± 138	315 ± 141	314 ± 133	.81
Sum of skinfold (mm)	11.8 ± 2.73	11.8 ± 2.25	11.9 ± 2.39	11.9 ± 2.17	.91
Adiposity (% fat)	9.54 ± 3.60	9.74 ± 3.25	9.58 ± 3.39	9.54 ± 3.11	.84
SGA	4(13.3)	48(8.7)	68(11.2)	17(7.3)	.23
LGA	0	39(7.0)	43(7.1)	19(8.1)	.45
Umbilical cord C peptide level at birth (μg/L)	0.90 ± 0.52	1.05 ± 0.63	1.00 ± 0.53	1.04 ± 0.62	.34
Apgar score at 5 minutes <7	0	1(0.2)	2(0.3)	1(0.4)	.92
Umbilical cord arterial pH < 7.0	0	1(0.2)	3(0.5)	2(0.8)	.58
Admission to neonatal ICU/special care unit	16(50.0)	244(42.1)	273(42.5)	96(38.9)	.60
**Ambient solar radiation at blood draw (OGTT) and neonatal vitamin D status**					
Ambient solar radiation (MJ/m^2^)	341 ± 46	384 ± 85	390 ± 72	411 ± 76	<.001
Umbilical cord serum total 25(OH)D at birth (nmol/L)	36.5 ± 18.9	37.5 ± 15.0	45.2 ± 19.1	50.4 ± 17.3	<.001
Umbilical cord serum to maternal serum 25(OH)D ratio	1.71 ± 0.82	0.96 ± 0.40	0.74 ± 0.33	0.57 ± 0.17	<.001

Continuous and categorical variables were expressed as mean ± SD and n (proportion in %), and were compared by ANOVA and chi-square tests, respectively.

Abbreviations: 25(OH)D, 25-hydroxyvitamin D; AUC_glu_, area under the curve of maternal glucose levels at the OGTT during pregnancy; BMI, body mass index; GDM, gestational diabetes mellitus; LGA, large for gestational age; OGTT, oral glucose tolerance test; SGA, small for gestational age.

Table 2 shows the maternal characteristics, and pregnancy and neonatal outcomes with umbilical cord serum total 25(OH)D levels; only 66(5.0%) neonates had sufficient vitamin D at birth. There were trends for increasing maternal age, maternal 2-hour glucose levels and decreasing maternal fasting glucose levels, body mass index (BMI) at delivery, maternal gestational weight gain, and primary cesarean section rate with higher umbilical cord serum total 25(OH)D levels. The rates of primary cesarean section due to failure to progress and fetal distress were not different among groups with different umbilical cord vitamin D status. Neonatal ICU/special care unit admission was significantly higher among those with higher umbilical cord serum total 25(OH)D level at ≥75 nmol/L when compared with those at the level between 50-74.9 nmol/L (*P* = .003). Umbilical cord serum total 25(OH)D level at birth increased as the ambient solar radiation at the time of birth (*P* = .01) and maternal serum total 25(OH)D level at midgestation (*P* < .001) increased.

**Table 2.** Comparisons of maternal characteristics, pregnancy, and neonatal outcomes by umbilical cord serum total 25(OH)D level

	Umbilical cord serum total 25(OH)D level (nmol/L)				*P*
	<25 (n = 138)	25-49.9 (n = 831)	50-74.9 (n = 286)	≥75 (n = 66)	
**Maternal characteristics at recruitment**					
Maternal age (years)	29.3 ± 5.3	30.7 ± 4.7	31.7 ± 4.5	31.4 ± 5.1	<.001
Parity					
0	75 (54.3)	503 (60.5)	163 (57.0)	49 (74.2)	.16
1	53 (38.4)	270 (32.5)	99 (34.6)	15 (22.7)	
≥2	10 (7.2)	58 (7.0)	24 (8.4)	2 (3.0)	
Prepregnant weight (kg)					
Prepregnancy BMI (kg/m^2^)	20.8 ± 3.5	20.7 ± 2.7	20.5 ± 2.6	20.1 ± 2.8	.28
Smoker, *n* (%)	5 (3.6)	18 (2.2)	4 (1.4)	1 (1.5)	.50
**Maternal characteristics at OGTT**					
Fasting plasma glucose level (mmol/L)	4.40 ± 0.30	4.37 ± 0.34	4.32 ± 0.30	4.28 ± 0.28	.01
1-hour plasma glucose level (mmol/L)	7.34 ± 1.57	7.62 ± 1.73	7.64 ± 1.58	7.35 ± 1.46	.18
2-hour plasma glucose level (mmol/L)	6.17 ± 1.09	6.59 ± 1.30	6.57 ± 1.33	6.32 ± 1.26	.002
AUC_glu_	757 ± 122	786 ± 136	785 ± 126	760 ± 118	.053
HbA1c (%)	4.97 ± 0.35	4.91 ± 0.36	4.93 ± 0.38	4.97 ± 0.28	.21
GDM	13(9.4)	109(13.1)	40(14.0)	6(9.1)	.45
**Maternal characteristics at delivery**					
Maternal BMI at delivery (kg/m^2^)	27.2 ± 4.0	26.9 ± 3.1	26.4 ± 2.8	26.1 ± 2.7	.01
Gestational weight gain (kg)	16.2 ± 4.5	15.5 ± 4.6	14.6 ± 4.1	14.8 ± 4.0	.003
**Pregnancy outcomes**					
Gestational age at delivery (week)	39.5 ± 1.4	39.4 ± 1.4	39.4 ± 1.4	38.9 ± 1.8	.02
Preterm delivery <37 week	7 (5.1)	33 (4.0)	11 (3.8)	6 (9.1)	.24
Preeclampsia	2(2.9)	15(1.8)	3(1.0)	2(3.0)	.49
**Primary cesarean section**	31 (22.5)	170 (20.5)	38 (13.3)	10 (15.2)	.03
Indication of primary cesarean section					
Failure to progress	5 (3.6)	31 (3.7)	6 (2.1)	1 (1.5)	.23
Fetal distress	8 (5.8)	36 (4.3)	13 (4.5)	3 (4.5)	
Other causes	18 (13.0)	103 (12.4)	19 (6.6)	6 (9.1)	
**Neonatal outcomes**					
Baby's gender (male)	72(52.2)	427(51.4)	157(54.9)	36(54.5)	.76
Birth weight (g)	3204 ± 431	3179 ± 411	3174 ± 403	3124 ± 408	.63
Fat free mass (g)	2900 ± 294	2868 ± 286	2865 ± 275	2872 ± 242	.67
Fat mass (g)	324 ± 139	321 ± 141	314 ± 129	312 ± 128	.84
Sum of skinfold (mm)	11.7 ± 2.4	11.9 ± 2.4	11.7 ± 1.9	11.7 ± 2.1	.43
Adiposity (% fat)	9.72 ± 3.16	9.71 ± 3.37	9.56 ± 3.05	9.53 ± 3.12	.91
SGA	10(7.6)	76 (9.5)	24 (8.7)	3 (5.0)	.62
LGA	11(8.4)	58 (7.3)	11 (4.0)	3 (5.0)	.21
Umbilical cord C peptide level at birth (μg/L)	1.05 ± 0.48	1.02 ± 0.48	0.97 ± 0.55	0.94 ± 0.55	.24
Apgar score at 5 minutes < 7	0	2(0.2)	1(0.3)	0	.88
Umbilical cord arterial pH < 7.0	0	2(0.2)	2(0.7)	1(1.5)	.27
Admission to neonatal ICU/special care unit	58(42.0)	337(40.6)	99(34.6)	36(54.5)	.02
**Ambient solar radiation at delivery and maternal vitamin D status**					
Ambient solar radiation at birth (MJ/m^2^)	368 ± 67	380 ± 75	394 ± 80	401 ± 73	.001
Maternal serum total 25(OH)D (nmol/L)	43.9 ± 16.8	55.8 ± 17.9	63.6 ± 20.2	66.4 ± 26.9	<.001
Umbilical cord serum to maternal serum 25(OH)D ratio	0.53 ± 0.18	0.73 ± 0.27	1.04 ± 0.42	1.62 ± 0.72	<.001

Continuous and categorical variables were expressed as mean ± SD and n (proportion in %), and were compared by ANOVA and chi-square tests, respectively.

Abbreviations: 25(OH)D, 25-hydroxyvitamin D; AUC_glu_, area under the curve of maternal glucose levels at the OGTT during pregnancy; BMI, body mass index; GDM, gestational diabetes mellitus; LGA, large for gestational age; OGTT, oral glucose tolerance test; SGA, small for gestational age.

### Factors Associated With Maternal and Umbilical Cord Serum Total 25(OH)D Level

**Figure 1.**
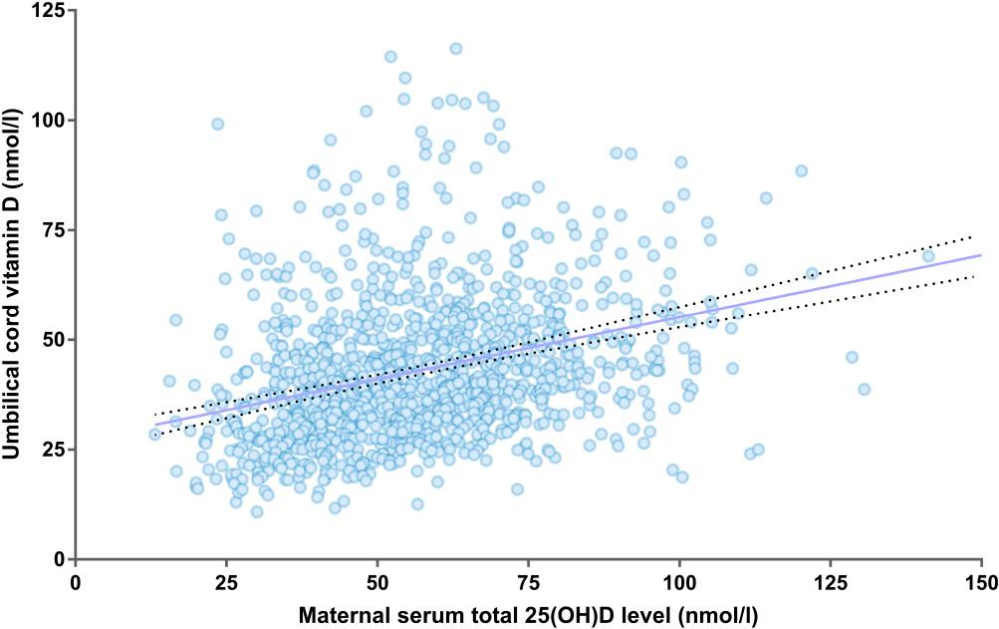
Scatter plot between maternal serum total 25(OH)D level and umbilical cord serum total 25(OH)D level. The solid line represents the trend line of a linear regression and the dotted lines represent its 95% CI.

**Figure 2.**
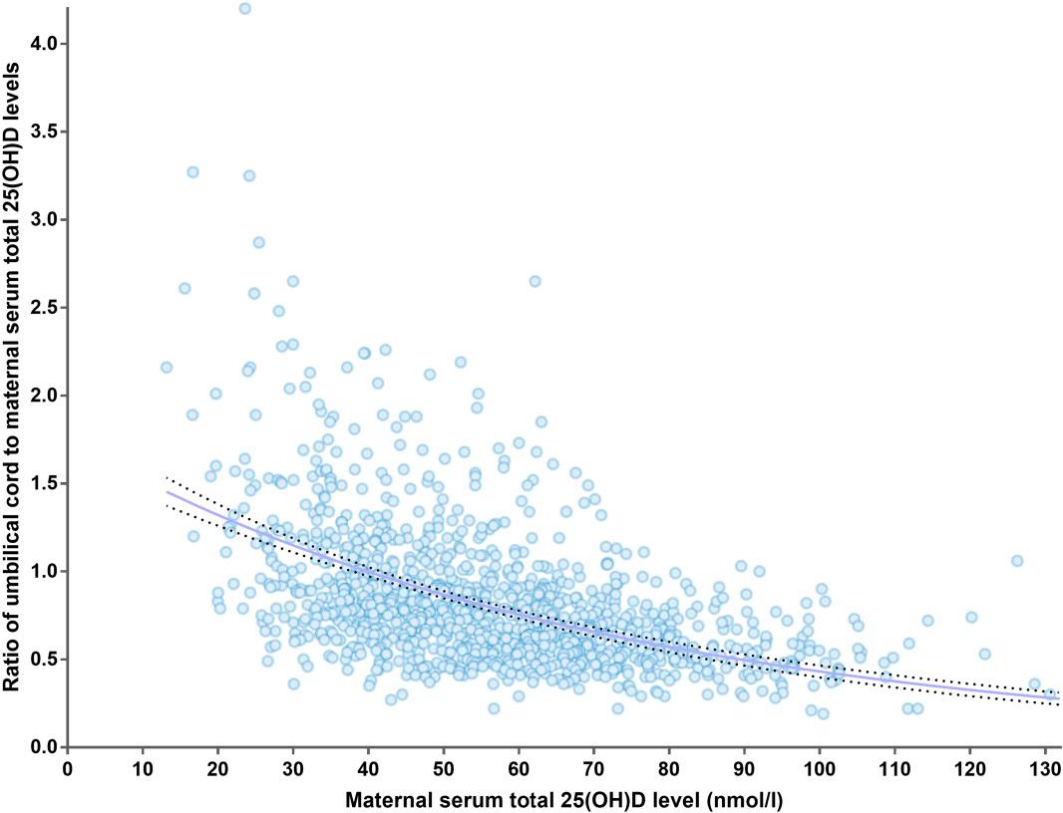
The log relationship between maternal serum 25(OH)D level and ratio of umbilical cord serum to maternal serum total 25 (OH)D. The trend line (solid line) is plotted with its 95% CI (dotted lines).

Figure 1 shows a significant linear association between the maternal serum total 25(OH)D levels and umbilical cord total serum 25(OH)D levels (goodness of fit R^2^ = 0.10, *P* < .001). Table 3 shows the multivariate logistic regression analyses for factors influencing the maternal and umbilical cord total 25(OH)D levels. After adjustment for potential confounders, maternal age and ambient solar radiation were positively associated, and maternal BMI was inversely associated with both maternal and umbilical cord serum 25(OH)D levels. Maternal GDM was associated with higher maternal 25(OH)D levels but not umbilical cord levels. Parity was positively associated with maternal 25(OH)D levels but negatively associated with the umbilical cord levels. Figure 2 shows the umbilical cord serum to maternal serum total 25(OH)D ratio, which may imply the transplacental vitamin D transfer, increases in a log scale with decreasing maternal serum 25(OH)D levels.

**Table 3.** Factors associated with maternal and cord serum total 25(OH)D level

	Maternal serum total 25(OH)D				Umbilical Cord serum total 25(OH)D			
	Univariate		Multivariate*^a^*		Univariate		Multivariate*^b^*	
	β (95% CI)	*P*	β (95% CI)	*P*	β (95% CI)	*P*	β (95% CI)	*P*
Maternal age (year)	0.005 (0.004 to 0.007)	<.001	0.005 (0.003 to 0.006)	<.001	0.005 (0.003 to 0.007)	<.001	0.005 (0.003 to 0.007)	<.001
Parity (≥1 vs 0)	0.038 (0.022 to 0.054)	<.001	0.016 (0.004 to 0.037)	.016	−0.011 (−0.030 to 0.007)	.230	−0.041 (−0.060 to −0.023)	<.001
GDM	0.04 (0.01 to 0.06)	.002	0.025 (0.002 to 0.047)	.032	0.013(−0.014 to 0.040)	.334	0.002(−0.025 to 0.028)	.899
Maternal BMI (kg/m^2^)	−0.003 (−0.005 to 0.000)*^c^*	.037	−0.005 (−0.007 to −0.002)*^c^*	<.001	−0.005(−0.008 to −0.002)*^d^*	.001	−0.003(−0.006 to 0.000)*^d^*	.034
Ambient solar radiation (per 10 MJ/m^2^)	0.003 (0.002 to 0.004)*^c^*	<.001	0.003 (0.002 to 0.004)*^c^*	<.001	0.003(0.002 to 0.004)*^d^*	<.001	0.003(0.002 to 0.004)*^d^*	<.001
Maternal serum total 25(OH)D (nmol/L)	—	—	—	—	0.003 (0.002 to 0.003)	<.001	0.003 (0.002 to 0.003)	<.001

Abbreviations: 25(OH)D, 25-hydroxyvitamin D; BMI, body mass index; β, unstandardized coefficient for log-transformed serum total 25(OH)D (nmol/L); GDM, gestational diabetes mellitus.

^*a*^Included maternal age, smoking status, parity, GDM, maternal BMI at blood draw, gestational age and ambient solar radiation at blood draw, and baby sex.

^*b*^Included maternal age, smoking status, parity, gestational diabetes mellitus, pre-eclampsia, maternal BMI at delivery, gestational age and ambient solar radiation at delivery, maternal serum total 25(OH)D level and baby sex.

^*c*^Refers to maternal BMI and the ambient solar radiation at the time of oral glucose tolerance test

^*d*^Refers to maternal BMI and the ambient solar radiation at the time of delivery

### Factors Associated With Ratio of Umbilical Cord to Maternal Serum Total 25(OH)D Level

Table 4 shows the multivariate logistic regression analyses on factors that influence the umbilical cord to maternal serum total 25(OH)D ratio. Maternal age and ambient solar radiation at delivery were positively associated, while parity, maternal serum total 25(OH)D at midgestation, and gestational age at birth were negatively associated with the umbilical cord to maternal serum total 25 (OH)D ratio.

**Table 4.** Factors associated with the ratio of umbilical cord to maternal serum 25 (OH)D

	Univariate		Multivariate	
	β (95% CI)	*P*	β (95% CI)	*P*
Maternal age (per 5 years)	−0.001 (−0.012 to 0.009)	.816	0.022 (0.012 to 0.032)	<.001*^a^*
Smoker	0.026 (−0.043 to 0.095)	.458	0.007 (−0.051 to 0.066)	.804*^a^*
Multiparity (parity ≥1 vs parity 0)	−0.048 (−0.069 to −0.027)	<.001	−0.041 (−0.060 to −0.021)	<.001*^a^*
GDM	−0.019 (−0.050 to 0.011)	.218	−0.006 (−0.032 to 0.021)	.683*^a^*
Maternal serum 25(OH)D (per 10 nmol/L)	−0.047 (−0.051 to −0.042)	<.001	−0.047 (−0.052 to −0.043)	<.001*^a^*
Preeclampsia	−0.025 (−0.101 to 0.051)	.520	−0.023 (−0.088 to 0.041)	.480*^a^*
Primary cesarean section	−0.008 (−0.032 to 0.016)	.523	−0.019 (−0.040 to 0.002)	.073*^a^*
Gestational age at birth (week)	−0.006 (−0.013 to 0.001)	.118	−0.007 (−0.013 to 0.000)	.037*^a^*
Maternal BMI at delivery (kg/m^2^)	−0.001 (−0.005 to 0.002)	.416	−0.002 (−0.005 to 0.000)	.094*^a^*
Ambient solar radiation at delivery (per 10 MJ/m^2^)	0.004 (0.003 to 0.005)	<.001	0.003 (0.002 to 0.005)	<.001*^a^*

Abbreviation: 25(OH)D, 25-hydroxyvitamin D; β, Unstandardized coefficient for the log-transformed ratio of umbilical cord serum to maternal serum 25(OH)D levels (nmol/L); AUC_glu_, area under the curve of maternal glucose levels at the oral glucose tolerance test; during pregnancy; GDM, gestational diabetes mellitus.

^*a*^Maternal age, smoking status, parity (≥1 vs 0), pre-eclampsia, primary cesarean section, gestational age and BMI at delivery, maternal serum 25(OH)D level, ambient solar radiation at the month of delivery, baby sex, GDM.

### Associations Between Maternal/Umbilical Cord Serum Total 25(OH)D Level and Pregnancy Implications and Neonatal Outcomes

Table 5 shows the association of both maternal and umbilical cord 25(OH)D levels with pregnancy and neonatal outcomes. Other than an association of lower primary cesarean section rate with umbilical cord serum total 25(OH)D (OR 0.990, 95% CI 0.982-0.999; *P* = .032), no other outcomes were significantly associated with either umbilical cord or maternal 25 (OH)D levels.

**Table 5.** The associations of serum 25(OH)D with pregnancy complications and neonatal outcomes

	Maternal serum 25(OH)D				Umbilical cord serum 25(OH)D			
	Univariate		Multivariate*^a^*		Univariate		Multivariate*^a^*	
	OR (95% CI)	*P*	OR (95% CI)	*P*	OR (95% CI)	*P*	OR (95% CI)	*P*
Preterm	1.005 (0.993-1.016)	.416	1.006 (0.994-1.018)	.361*^a^*	1.013 (1.001-1.024)	.033	1.008 (0.996-1.021)	.183*^a^*
Preeclampsia	1.006 (0.989-1.023)	.504	1.007 (0.989-1.026)	.437*^b^*	—	—	—	—
Primary cesarean section	0.993 (0.986-0.999)	.028	0.994 (0.986-1.001)	.091*^c^*	0.993 (0.985-1.001)	.101	0.990 (0.982-0.999)	.032*^c^*
SGA	0.996 (0.987-1.005)	.415	0.998 (0.988-1.007)	.625*^c^*	0.997 (0.986-1.009)	.638	0.995 (0.983-1.007)	.430*^c^*
LGA	1.001 (0.991-1.012)	.790	1.003 (0.992-1.015)	.541*^c^*	0.991 (0.977-1.006)	.232	0.996 (0.980-1.011)	.570*^c^*
Adiposity (% fat ≥90th percentile)	1.003 (0.994-1.012)	.506	1.006 (0.996-1.016)	.232*^c^*	1.004 (0.993-1.014)	.498	1.010 (0.999-1.022)	.082*^c^*
Apgar score at 5 minutes < 7	1.016 (0.973-1.061)	.473	0.969 (0.896-1.048)	.429*^c^*	0.995 (0.929-1.065)	.876	0.006 (0.000-3.57e21)	.855*^c^*
Umbilical cord arterial pH < 7.0	1.016 (0.980-1.053)	.387	1.017 (0.980-1.056)	.368*^c^*	1.025 (1.002-1.048)	.033	1.028 (0.995-1.063)	.102*^c^*
Admission to neonatal intensive care unit	0.998 (0.992-1.003)	.356	0.998 (0.992-1.003)	.409*^c^*	1.005 (0.999-1.012)	.077	1.003 (0.996-1.009)	.403*^c^*

Abbreviations: 25(OH)D, 25-hydroxyvitamin D; BMI, body mass index; GDM, gestational diabetes mellitus.

^*a*^Adjusted by maternal age, multiparity, BMI at delivery, GDM status, preeclampsia, ambient solar radiation, smoking, and sex of baby.

^*b*^Adjusted by maternal age, multiparity, BMI at delivery, GDM status, ambient solar radiation, smoking, sex of baby, and gestational age at delivery.

^*c*^Adjusted by maternal age, multiparity, BMI at delivery, GDM status, preeclampsia, ambient solar radiation, smoking, sex of baby, and gestational age at delivery.

After subgroup into different indications, umbilical cord serum total 25(OH)D was persistently associated with lower risk of primary cesarean section due to failure to progress (OR 0.973, 95% CI 0.950-0.997; *P* = .025) and other causes (OR 0.989, 95% CI 0.978-1.000; *P* = .044). (Table 6)

**Table 6.** Associations of maternal and umbilical cord serum 25(OH)D with cesarean section due to different indications

Indication for primary cesarean section	Maternal serum 25(OH)D				Umbilical cord serum 25(OH)D			
	Univariate		Multivariate*^a^*		Univariate		Multivariate*^a^*	
	OR (95% CI)	*P*	OR (95% CI)	*P*	OR (95% CI)	*P*	OR (95% CI)	*P*
Failure to progress	0.993 (0.979-1.008)	.368	0.993 (0.977-1.010)	.440	0.980 (0.959-1.002)	.072	0.973 (0.950-0.997)	.025
Fetal distress	0.992 (0.979-1.004)	.189	0.992 (0.979-1.005)	.240	1.004 (0.990-1.018)	.585	1.001 (0.986-1.016)	.931
Other causes	0.994 (0.985-1.002)	.161	0.995 (0.985-1.004)	.285	0.992 (0.981-1.002)	.130	0.989 (0.978-1.000)	.044

Abbreviations: 25(OH)D, 25-hydroxyvitamin D; BMI, body mass index; GDM, gestational diabetes mellitus.

^*a*^Adjusted by maternal age, multiparity, BMI at delivery, GDM status, preeclampsia, ambient solar radiation, smoking, sex of baby, and gestational age at delivery.

## Discussion

Among a population with high prevalence of vitamin D deficiency, maternal age, BMI, and ambient solar radiation are factors influencing vitamin D levels in mothers and their neonates. The placental transfer of vitamin D was found to be positively associated with maternal age and ambient solar radiation at the time of delivery, while negatively associated with maternal serum total 25(OH)D at midgestation. The associations of maternal and umbilical cord 25(OH)D levels with adverse pregnancy and neonatal outcomes remain uncertain.

In line with previous reports among Western [19, 20] and Chinese populations [21], this study observed maternal age and ambient solar radiation were positively associated with both maternal and umbilical cord 25(OH)D levels. The inverse associations between maternal BMI and 25(OH)D levels were similar in mothers and infants. As vitamin D is a fat-soluble molecule sequestered in the larger adipose pool in obese individuals [22], it is expected that fetal 25(OH)D would be lower in the mothers of higher BMI as the source of vitamin D is from the mother.

The expression of the vitamin D receptor (VDR) in pancreatic beta cells suggests the involvement of vitamin D in insulin production, but the evidence for the association of maternal hyperglycemia with vitamin D status during pregnancy varied. Some earlier studies reported inverse associations between GDM and maternal vitamin D [5, 23], while reports from HAPO Study cohorts did not observe an association [19, 24]. In contrast, our study found a positive association between GDM and maternal vitamin D levels. Similarly, an earlier study also reported higher maternal vitamin D levels among pregnant women with abnormal glucose challenge test [25]. Of note, the sera for the maternal vitamin D assay were fasting samples collected at the time of OGTT in midgestation; thus, a temporal association of the onset of GDM with early pregnancy vitamin D status could not be demonstrated in the current study. In addition, details of dietary intake and vitamin supplementation were not collected. Given the above, the association between GDM and maternal vitamin D status needs further clarification.

Our study also examined the factors influencing the placental transfer of vitamin D and found that the gradient was positively impacted by maternal age and ambient solar radiation and negatively affected by parity, maternal vitamin D levels and the length of gestation. It was traditionally thought that vitamin D simply diffused from the maternal circulation to the fetus via the placenta depending on the concentration difference. However, a recent study demonstrated that maternal vitamin D was actively and selectively transferred to the placenta, which modulated the amount of vitamin D transported to the fetus or stored and metabolized in situ [26]. The active form of vitamin D, 1,25(OH)D, performs its biological functions by binding to the VDR and interacting with the retinoid-X receptor. The elevated levels of VDR/retinoid-X receptor in the placenta increase the amount of enzymes that break down 25(OH)D and 1,25(OH)D (eg, CYP24A1) and therefore decrease the amount of vitamin D transferred to the fetus [26]. Compared with young controls, VDR expression was markedly decreased in the placenta of older mice [27]. In this study, increased maternal age and prolonged sun exposure were positively associated with the placental transfer of vitamin D to the fetus. It may be related to decreased VDR expression in the placenta of older pregnant women, which consequently enhanced the amount of vitamin D transferred from the mother to the fetus. When the maternal vitamin D is low, the expression of CYP24A1 is suppressed to increase the transport of vitamin D via the placenta to support the fetus [26]. Of note, it was reported that the vitamin D-binding protein (VDBP) increased longitudinally across the pregnancy, and the free 25(OH) levels decreased as a consequence [28]. The VDBP polymorphism may also affect the VDBP and vitamin D status. For instance, the minor allele for rs7041 was associated with decreased VDBP and increased total vitamin D levels [29]. In another work, among the cases with similar vitamin D status, those with lower VDBP expressions in placenta theoretically have greater amount of free 25(OH)D transport to the fetus. In this study, we have no information about the genotypes of the VDBP gene and the VDBP levels. Future studies are warranted to confirm whether placental transfer of vitamin D is mediated by active mechanism or determined by the VDBP polymorphism.

We also examined the association of maternal and umbilical cord serum vitamin D levels with adverse pregnancy and neonatal outcomes. It has been postulated that sufficient vitamin D is critical for maintaining skeletal and smooth muscle strength during labor by regulating calcium homeostasis. Women with vitamin D <37.5 nmol/L were reported to have nearly 4 times the risk of primary cesarean section compared with women with higher vitamin D levels [30]. This was supported by Rodriguez and colleagues as they found the risk of nonelective, nonemergency cesarean section was reported to be 40% lower in women with sufficient vitamin D (25(OH)D > 75 nmol/L) than in those deficient in vitamin D (<50 nmol/L) [7]. But these associations were not consistent with findings from other investigators [10, 31]. In our study, we observed an association between higher umbilical cord serum 25(OH)D level and lower risk of primary cesarean section due to failure to progress and other causes except fetal distress. However, the magnitude of the effect of vitamin D on the cesarean section rate was small, and the rate of cesarean section showed no significant difference in the common indications for intrapartum cesarean section. Thus, the impact of vitamin D levels on the risk of cesarean section needs further investigation.

We observed no associations between vitamin D levels and preterm delivery, preeclampsia, and fetal anthropometry and morbidity. Vitamin D was previously reported to participate in the regulation of innate and acquired immune responses in the placenta [32, 33]. Compared with term delivery, the inflammatory biomarkers S100A8, HMGB1, TLR2, and NF-kappaB were elevated in preterm placental tissue, and the amount of the inflammatory protein was negatively correlated with umbilical cord vitamin D levels [34]. Furthermore, the antimicrobial peptides cathelicidin and LL-37 were downregulated in the placentas with decreased VDR expression [34]. The anti-inflammatory and anti-infectious functions of vitamin D suggest that vitamin D deficiency during pregnancy might contribute to the increased risk of preterm birth, as summarized in some meta-analyses [35, 36]. However, there were notable case–control studies included in the meta-analysis that may overestimate the effect size of the associations, and the heterogeneous definitions of vitamin D and preterm birth add to the difficulties in interpreting these findings. In agreement with the findings from a previous cohort study [7] and randomized control trial [31], our results from a prospective cohort provide no evidence for the association of vitamin D status with the risk of preterm delivery.

Preeclampsia and SGA are considered placenta-derived disorders. Regardless of the immunomodulatory role that vitamin D may exert on early placental development, the association of vitamin D deficiency with preeclampsia and SGA remains inconclusive. Serum 25(OH)D levels in the women with preeclampsia were similar to the healthy controls [37]. Likewise, our study did not observe an association between vitamin D status and the risk of preeclampsia and SGA, which was in contrast to the findings from some previous meta-analyses. It was noted that the pooled estimate in a meta-analysis increased when stratified by 25(OH)D >75 nmol/L, sampling time at midgestation or late gestation, case–control study design, and 25(OH)D measured by an immunoassay method. A recent study also showed that maintaining 25(OH)D >75 nmol/L from early to late pregnancy resulted in a lower risk of preeclampsia [38]. However, the majority of participants in our study had maternal and umbilical cord serum 25(OH)D levels below 75 nmol/L. Thus, our study may not have had sufficient power to detect an association between vitamin D status and adverse outcomes due to the small number of vitamin D sufficient cases. Furthermore, using the liquid chromatography–tandem mass spectrometry method to measure 25(OH)D levels and thereby distinguish biologically inactive forms of metabolites could have prevented missing a diagnosis of suboptimal vitamin D status, as we previously reported [11]. Neither maternal levels at midgestation nor fetal levels at birth were associated with the risk of NICU admission in this study. We observed a decreasing trend of NICU admission with higher umbilical cord 25(OH)D levels, but admissions dramatically increased to 54.5% when umbilical cord 25(OH)D was ≥75 nmol/L. The indications for NICU admission varied from acute fetal distress and severe maternal complications to fetal congenital anomalies, but the indications for admission were not available in the current study. The associations of vitamin D status with neonatal intensive care unit admission are still unclear.

The present study was based on a large prospective cohort study that provided objective evidence on the association of vitamin D status with pregnancy and neonatal outcomes. Moreover, vitamin D levels were measured using the current golden-standard assay method. However, there were some limitations in this study. We have maternal sera to measure vitamin D levels only at midgestation, which may not represent the overall vitamin D status throughout the pregnancy. The most critical period for the impact of vitamin D on pregnancy and neonatal outcomes is still uncertain. Longitudinal studies with repeated measurement of vitamin D during pregnancy are warranted to confirm the association. Furthermore, we did not measure the levels of VDBP, free 25(OH)D and 1,25(OH)D in this study. Knowing the status of these metabolites provides more information about the factors influencing the placental transfer and the associations with pregnancy and neonatal outcomes. In addition, this study did not collect data regarding dietary intake, prenatal vitamin supplementation, and physical exercise intensity, all of which may affect maternal wellness and fetal growth.

### Conclusion

We observed suboptimal vitamin D status in pregnant women and their neonates in this cohort. Placental vitamin D transfer appeared to be greater in the presence of low maternal vitamin D level, suggesting an active transport mechanism to compensate. Older maternal age and ambient solar radiation also have greater transplacental transport of vitamin D to the fetus. The protective effect of sufficient vitamin D in a cesarean section will require further studies.

## Data Availability

Restrictions apply to the availability of some or all data generated or analyzed during this study to preserve patient confidentiality or because they were used under license. The corresponding author will on request detail the restrictions and any conditions under which access to some data may be provided.

## Abbreviations

25(OH)D: 25-hydroxyvitamin D
ANOVA: analysis of variance
BMI: body mass index
GDM: gestational diabetes mellitus
HAPO: Hyperglycemia and Adverse Pregnancy Outcome
LGA: large for gestational age
OGTT: oral glucose tolerance test
SGA: small for gestational age
VDBP: vitamin D-binding protein
VDR: vitamin D receptor
